# In vitro antioxidant, anti-inflammatory, and anticancer activities of mixture Thai medicinal plants

**DOI:** 10.1186/s12906-023-03862-8

**Published:** 2023-02-10

**Authors:** Suchada Jongrungraungchok, Fameera Madaka, Thaniya Wunnakup, Teeratad Sudsai, Chanamon Pongphaew, Thanapat Songsak, Nalinee Pradubyat

**Affiliations:** 1 Department of Pharmaceutical Chemistry, College of Pharmacy, Rangsit University, Pathum Thani, 12000 Thailand; 2 Drug and Herbal Product Research and Development Center, College of Pharmacy, Rangsit University, Pathum Thani, 12000 Thailand; 3 Princess Srisavangavadhana College of Medicine, Chulabhorn Royal Academy, Bangkok, 10210 Thailand; 4 Department of Pharmacognosy, College of Pharmacy, Rangsit University, Pathum Thani, 12000 Thailand; 5 Department of Pharmacology, College of Pharmacy, Rangsit University, Pathum Thani, 12000 Thailand

**Keywords:** Antiproliferative activity, Antimigration, Alpinia galanga, Piper nigrum, Citrus aurantifolia, Tiliacora triandra, Cannabis sativa

## Abstract

**Background:**

The phytochemical study of medicinal plants is rapidly gaining popularity with many pharmacologic effects. This study aims to determine the antioxidant capacity as well as anticancer and antimigration activities of Clear belongs Plus extract (CBL-P) which consisted of five medicinal plants namely, *Alpinia galanga*, *Piper nigrum*, *Citrus aurantifolia*, *Tiliacora triandra*, and *Cannabis sativa* on human colon cancer cells SW620 and HCT116 cell lines, and human non-small cell lung cancer cells A549 and NCI-H460 cell lines.

**Methods:**

In this study the dried-plant powder was extracted using 90% ethanol. Additionally, CBL-P was studied antioxidative activity via DPPH and ABTS assays and anti-inflammatory activities using nitric oxide assay using Griess reaction. Antiproliferation and antimigration of CBL-P were investigated using 3-(4,5-dimethylthiazol-2-yl)-2,5-diphenyl-2H-tetrazolium bromide (MTT) and scratch assay.

**Results:**

The results showed that CBL-P had potent antiproliferative activity with IC_50_ values in a concentration- and time-dependent manners for all four cell lines. CBL-P also possessed potent antimigration activity against all studied cancer cells. CBL-P demonstrated antimigration activity on four different types of cancer cells (A549, NCI-H460, HCT116, and SW620) after 48 h of incubation, with the greatest effect seen at the highest concentration tested (15 μg/mL) in A549 cells (10.23% of wound closure) and NCI-H460 cells (9.16% of wound closure). CBL-P was also effective in reducing migration in HCT116 and SW620 cells, with a range of closure area from 10—50%. In addition, CBL-P had antioxidant activity with IC_50_ values of 8.549 ± 0.241 mg/mL and 2.673 ± 0.437 mg/mL for DPPH and ABTS assays, respectively. CBL-P also showed anti-inflammatory activity with the best inhibitory activity on NO production at a concentration of 40 μg/mL.

**Conclusion:**

In conclusion, the mixture extract possessed antioxidant and anti-inflammatory activity. Furthermore, the mixture plant extract significantly exhibited antiproliferative and antimigration activities on SW620, HCT116, A549, and NCI-H460 cells (*P* ≤ 0.05). Taken together, our results suggest that medicinal plants may have synergistic effects that could potentially enhance the effectiveness of cancer treatment when used as adjuvants. These findings provide a solid scientific foundation for future efforts to explore the mechanism of action.

## Background

Antioxidants generated from plants are a vast category of naturally occurring substances having reducing or radical-scavenging properties [1]. Antioxidant, anti-inflammatory, antibacterial, and anticancer activity are some of the biological and medicinal qualities attributed to these plant metabolites [1, 2]. Many phenolic chemicals, such as flavonoids, tannins, and curcumins, are believed to exert their effects by scavenging free radicals or inhibiting pro-inflammatory enzymes such as cyclo-oxygenases (COX) and lipoxygenases (LOX) in the inflammatory cascades [3, 4]. It is believed that oxidative stress plays a significant role in the aetiology of cardiovascular illnesses, neurodegeneration, malignancies, immunological disorders, diabetes, ageing, and other conditions [5–7]. Plants, mainly fruits and vegetables, are an abundant source of antioxidants [1, 6, 7]. It is hypothesised that antioxidants provide health advantages by reducing oxidative stress directly [1]. The body's antioxidant network functions through various methods, including ROS scavenging, lipid peroxidation termination, and metal chelation [1, 8]. Despite the extensive information about antioxidant structures, characteristics, and biological effects that had been accumulated, many elements required clarification and more research [1]. Nitric oxide (NO) is a free radical with a short half-life that mediates several biological activities. NO enhances the bactericidal and tumoricidal actions of activated macrophages as one of its roles [1]. However, excessive NO generation may result in tissue injury and activation of pro-inflammatory mediators [3]. It has been established that extracts from medicinal plants may scavenge these free radicals and control inflammatory responses [1, 3].

Lung cancer and colon cancer are among the most common types of cancer globally [9]. Non-small cell lung cancer is the most common form of lung cancer and accounts for approximately 85% of all cases [10]. There are three main types of non-small cell lung cancer: adenocarcinoma, large cell carcinoma, and squamous cell carcinoma. Adenocarcinoma is the most prevalent form of lung cancer in the United States and typically originates in the outer sections of the lungs, while large cell carcinomas are characterised by large, abnormal-looking cells that can develop anywhere in the lungs and tend to grow rapidly, and squamous cell carcinoma typically starts in the bronchi near the centre of the lungs. In this study, two non-small cell lung cancer cell lines (A549 and NCI-H460) were selected to be tested for their response to the extract [11]. A549 is a cell line model of non-small cell lung cancer used to evaluate the role of specific proteins in cancer growth and spread, while NCI-H460 cells have a faster growth rate compared to A549 cells [11]. Colorectal cancer, also known as colon cancer, is the third most common type of cancer globally [9]. Adenocarcinoma is the most common type of colorectal cancer, comprising 95% of all cases. HCT116 and SW620 are human colorectal carcinoma cell lines known for their high aggressiveness [12]. Several genes, including APC, TP53, ERBB2, KRAS, PTEN, and BRAF, are commonly altered in colorectal cancer and can contribute to the development and progression of the disease [13, 14]. The four cell lines selected for this study (HCT116, SW620, A549, and NCI-H460) are frequently used in research as model systems for human colon cancer and non-small cell lung cancer.

Thailand is home to various natural plants; making it ideal for the exploration of phytochemical compounds and its medicinal properties [15, 16]. More than 50% of cancer chemotherapeutic drugs are derived from pure or chemically altered natural plant extract, but only about 15% have been tested for bioactive compounds making this an under-explored field [17]. There is a growing interest in traditional medicine as a source of new therapies, but the scientific evidence supporting these practices is often limited [17]. Many traditional medicine practices are based on centuries of empirical observation and are supported by cultural and anecdotal evidence, but have not been rigorously tested using modern scientific methods [18, 19]. This is particularly true for plant-based therapies, which have a long history of use in traditional medicine but have not always been subjected to rigorous scientific investigation [18]. Clear belongs Plus extract (CBL-P) consisted of five medicinal plants namely, *Alpinia galanga*, *Piper nigrum*, *Citrus aurantifolia*, *Tiliacora triandra*, and *Cannabis sativa*. *A. galanga* is a plant in the ginger family that is native to Southeast Asia [20]. It is commonly used in traditional medicine for its anticancer, anti-inflammatory, analgesic, and antibacterial properties [21, 22]. *P. nigrum* is a plant commonly known as black pepper and is native to tropical regions of Asia [23]. It is commonly used as a spice and has been shown to have antioxidant, anti-inflammatory, and anticancer properties [24]. *C. aurantifolia*, also known as lime, is a plant in the citrus family and is native to Southeast Asia [25]. It is commonly used as a food and in traditional medicine for its antihypertensive activity, antiviral, antibacterial, and antioxidant properties [25, 26]. *T. triandra* is a plant in the family Menispermaceae that is native to Southeast Asia [27]. It has traditionally been used in traditional medicine for its antihypertensive activity, anti-inflammatory and antipyretic effects [26–28]. *C. sativa* is a plant in the family Cannabaceae that is native to Central and South Asia [29]. It is commonly known for its psychoactive effects and has been used in traditional medicine for its anticancer, anti-inflammatory, analgesic, and anti-anxiety properties [30, 31]. The selection of *A. galanga*, *P. nigrum*, *C. aurantifolia*, *T. triandra*, and *C. sativa* for this study was based on their traditional use and medicinal properties.

The use of plant-based therapies, particularly those found in traditional medicine, is becoming increasingly popular as people seek more natural and holistic approaches to health and wellness [18]. However, the scientific evidence supporting these practices is often limited, and there is a need for more research to build solid scientific evidence for traditional medicine practices, including the use of plant extracts [18]. Hence, this study aims to investigate the potential anticancer effects of a mixture extract containing these five plants, known as Clear belongs Plus extract (CBL-P), in human cancer cell lines. In particular, the study focused on the antiproliferative and antimigration activities of CBL-P in human colorectal cancer (SW620, HCT116 cell lines) and human non-small cell lung cancer cells (A549, and NCI-H460 cell lines). Additionally, the antioxidant and anti-inflammatory activities of CBL-P were also evaluated, as these properties may contribute to its potential as an anticancer agent. This study contributes to the growing body of scientific evidence on the potential therapeutic benefits of traditional medicine practices and highlights potential therapeutic benefits of traditional medicine practices.

## Methods

### Plants collection

Various regions of Thailand were sourced for plant materials. The *Alpinia galangal* (rhizome), *Piper nigrum* (seed), *Citrus aurantifolia* (lime peel), *Tiliacora triandra* (leaves), and *Cannabis sativa* (leaves) were obtained during October 2021 and cultivated in Bangkok, Thailand. The collection method was optimum for conditions for regeneration of the plants. All methods were carried out in accordance with the standard guidelines of Living Collection Policy of the Botanical Garden Organization, Ministry of Natural Resources and Environment, Thailand. The plants were identified by Assistant Professor Thanapat Songsak and plant materials were kept at the herbarium of the Department of Pharmacognosy, College of Pharmacy, Rangsit University, Thailand, where the herbarium. The given voucher specimen number for *A. galangal*, *P. nigrum*, *C. aurantifolia*, *T. triandra*, and *C. sativa* were CC-Ag-125, NV-Pn-136, PT-Ca-122, NV-Tt-138, and TS-Cs-21–134, respectively.

### Preparation of ethanolic extract of the plants

The plant materials were cleaned with tap water to eliminate extraneous impurities and chopped into small pieces, oven-dried at 50 °C until dry weight stability was observed, and then ground using an electric grinder. Three days were spent macerating 90 g of powdered plant materials (*A. galangal: C. aurantifolia: T. triandra*: *C. sativa: P. nigrum* in ration of 1:1:1:1:0.5) in 500 millilitres of 90% ethanol at room temperature (25–30 degrees Celsius). The ratio of *A. galangal: C. aurantifolia: T. triandra: C. sativa: P. nigrum* in this study was based on the traditional use of CBL-P in Thai traditional medicine. This particular ratio has been used in previous studies and has been shown to have various biological activities. The specific ratio of these plants was chosen in order to maximise the potential therapeutic benefits of the CBL-P extract. In addition, the use of this specific ratio allows for the investigation of any potential synergistic effects between the different plants in the mixture extract.

The extracted solvent was separated and filtered via Whatman no. 1 filter paper (Merck). The process was repeated for three times and then the filtrates were pooled together. Following filtering, the extracts were rotary-evaporated at decreased pressure. The plant-mixture extract or Clear belongs Plus extract (CBL-P) was kept at -20 degrees Celsius until use. The use of 90% ethanol in this study was chosen because it is a relatively high concentration of ethanol, which may result in a more efficient extraction of compounds from the plant material [32]. In South-East Asia, alcohol is often used as a base for herbal medicines [32]. Herbs are believed to be more effective and work faster when prepared in alcohol rather than water [32]. Additionally, it has been shown to be effective in the extraction of specific compounds from certain plant species, particularly those that are highly resinous, such as ginger and galangal [33]. Ginger requires 90% ethanol as a solvent, and lower quality extracts contain fewer pungent principles [33]. Furthermore, ethanol is a commonly used solvent for the preparation of plant extracts because it is able to dissolve a wide range of compounds found in plants, including alkaloids, flavonoids, tannins, and saponins [32]. It is also relatively inexpensive and easy to obtain, making it a popular choice for extract preparation [34]. Therefore, the use 90% ethanol as the solvent in this study was based on its ability to effectively extract a wide range of compounds from the plant material, as well as its cost-effectiveness and ability to sterilise the plant material before extraction.

### Preparation of plant mixture extract and reference drug

The CBL-P was dissolved in dimethyl sulfoxide (DMSO) to prepare a stock solution (50 mg/mL). The working solution was prepared from concentrated stock solution, and studied concentrations were prepared by serial dilution (1:2) with a complete medium. In each experiment, the plant extract was dissolved in dimethyl sulfoxide (DMSO) as a solvent, and the final concentration of DMSO in the complete medium was 0.2%.

### In vitro studies

#### Determination of antioxidant activity by DPPH assay

The antioxidant potential of CBL-P was measured by using diphenylpicrylhydrazyl (DPPH) radical scavenging technique. CBL-P at six different concentrations (0—400 μg/mL) were added in triplicate to a 0.1 mL solution of DPPH (Sigma-Aldrich, St. Louis, MO, USA) in ethanol in a 96-well microplate, which was then covered in aluminium foil and incubated at 37 °C for 30 min. At 517 nm, spectrophotometric measurements were done using a Bio-Rad Benchmark Plus Microplate Spectrophotometer. The radical scavenging activity was reported as a percentage of antioxidant activity using the following formula.

$$\mathrm{Antioxidant\;activity} \left(\%\right) = \frac{{\mathrm{A}}_{\mathrm{control}- }{\mathrm{A}}_{\mathrm{sample }}}{{\mathrm{A}}_{\mathrm{control}}} \times 100$$

From the formula, A_control_ = absorbance of the control sample and A_sample_ = absorbance of the test sample. Ascorbic acid was used as a positive control. The 50% inhibitory concentration (IC_50_) values (sample concentration required to scavenge 50% DPPH free radicals) were derived from a simple regression analysis.

#### Determination of antioxidant capacity by ABTS assay

CBL-P was also measured antioxidative capability by using 2,2'-azino-bis(3-ethylbenzothiazoline-6-sulfonic acid) (ABTS) assay. The preparation of Radical ABTS^•+^ was promoted by potassium persulfate to cause an oxidation of ABTS. ABTS (7 mM) and potassium persulfate (4.95 mM) were mixed and prepared as equal proportion and stored at room temperature in the dark for 16 h. After that ABTS/potassium persulfate mixture was diluted with methanol to achieve the absorbance values in range of 1 and 1.5 at 734 nm. Then 0.1 mL of the CBL-P extract at 25, 50, 100, 200, and 400 μg/mL was added 3.9 mL of the diluted ABTS^•+^. The reduction in absorbance was observed at 734 nm using UV- spectrophotometer. ABTS^•+^ was used as a blank and Trolox was a positive control in this investigation.

#### Inhibition of nitric oxide (NO) production

The mouse leukemic macrophage cell line (RAW 264.7) was obtained from the American Type Culture Collection (Rockville, MD, USA) and was maintained with complete medium (RPMI1640 supplemented with 10% FBS and 1% pen/strep). Cells were seeded in 96 well plates (1 × 10^5^ cell/well) and were activated with LPS (1 g/mL) in a complete medium and various concentrations (0 – 160 μg/mL) of CBL-P dissolved DMSO.

The nitric oxide production from macrophages was evaluated by determining the nitrite level in the culture medium using the Griess reagent. After the treatment of CBL-P for 24 h, culture medium (100 μL) was taken from each well and then Griess reagent (100 μL) was added. The mixture was then incubated for 10 min and measured the absorbance at the wavelength 550 nm by using a microplate reader (Bio-Rad Benchmark Plus Microplate Spectrophotometer). The nitrite levels were observed using regression analysis. The percentage inhibition of NO production was determined by comparing CBL-P-treated cell with LPS-treated control (cells without CBL-P), which was regarded to have no effect on inhibition of NO production.

To confirm that the suppression of NO production of CBL-P was not related to harmful effects to macrophage cells, the cytotoxicity was also parallelly conducted, as previously described by Mosmann, with minor changes [35]. After removing the existent medium, fresh complete medium (100 μL) was added to the cells. After that 10 μL of MTT solution (5 mg/mL) was added to each well to get the final concentration of MTT solution at 0.45 mg/mL. The cells were incubated with 5% CO_2_ at 37 °C for 4 h. After that the medium was removed, and the resulting formazan salt was dissolved in DMSO and the absorbance was measured at 570 nm using spectrophotometer. The percentage of cell viability was determined relative to the non-treated control (cells without LPS-containing extracts were considered to have 100 per cent viability). All tests measuring the inhibition of nitric oxide were completed three times in duplicate.

#### Human cell lines maintenance

SW620, HCT116, A549, and NCI-H460 cell lines were obtain from the American Type Culture Collection (ATCC) and cultured in RPMI-1640 medium supplemented with 10% Fetal bovine serum (FBS), 100 U/ml pen/strep and incubated at 37 °C in humidified air with 5% carbon dioxide. The medium was changed every two days. For additional in vitro studies, the cell lines were subcultured at 80% confluence and passaged onto 96 well plates (5000 cells/well) or 6 well plates (200,000 cells/well).

#### Cytotoxic and antiproliferative activities testing

SW620, HCT116, A549, and NCI-H460 cell lines 100 μL of cell suspension (5 × 10^5^cells/mL) was grown in RPMI1640 complete medium. Cell viability was assessed using a previously established methylthiazolyldiphenyltetrazolium bromide (MTT) test [36]. Each well of a 96-well plate was seeded with 5 10^5^ cells/mL and incubated for 24, 48, and 72 h. The cells were exposed to CBL-P at 10 to 320 μg/mL. After 24, 48 and 72 h of the treatment, the medium was replaced with MTT solution (0.5 mg/mL) and incubated at 37 °C in the dark for 4 h. The purple formazan crystals were dissolved using 100 μL DMSO and measured the absorbance at 570 nm using a microplate reader for further determination of the antiproliferative activity [37]. The results were reported as 50% inhibitory concentration (IC_50_). The calculation of the percentage cell viability as shown below:

$$\frac{\mathrm{Mean\;absorbance\;of\;treated\;cells}}{\mathrm{Mean\;absorbance\;of\;untreated\;cells}} \times 100$$

#### Antimigration assay using would healing assay

SW620, HCT116, A549, and NCI-H460 cell lines were cultured on 6-well plate at 37 °C in humidified air and 5% CO_2_ until 100% confluence. Using a plastic pipette diameter of 0.1 mm, gently and slowly scratch across the centre of the well. Then, wash the cells twice with a serum-free medium. After that, the cell lines were treated with CBL-P at 3.75, 7.5 and 15 μg/mL for 48 h. The time points chosen for the antimigration evaluation in a particular study may depend on a variety of factors, including the specific aims of the study, the type of cancer cells being used, and the expected time course of cell migration. In this specific study, the evaluation was conducted at 0 and 48 h because these time points were sufficient to observe any changes in migration. The wound width was measure using an inverted microscope at 5 × magnificent at 0 and 48 h. The space from scratch treatment between the control and treatment culture cell was quantified using Image J software to determine percentage of wound closure [37]. Calculation of total surface area, migrated cell surface area, and percentage of wound closure were calculated by the formula below.

$$\mathrm{Total\;surface\;area}=\mathrm{width\;of\;wound}\left(\mathrm{mm}\right) \times \mathrm{lenght\;of\;wound }\left(\mathrm{mm}\right)$$

$$\mathrm{Migrated\;cell\;surface\;area}=\mathrm{lenght\;of\;cell\;migration}\left(\mathrm {mm}\right) \times 2\mathrm{\;x\;lenght\;of\;wound}\left(\mathrm{mm}\right)$$

$$\mathrm{Percentage\;of\;wound\;closure}= \frac{\mathrm{Migrated\;cell\;surface\;area}}{\mathrm{Total\;surface\;area}} \times 100\%$$

### Measuring total phenolic and flavonoid contents

To determine the total phenolic content (TPC) of a sample, the Folin-Ciocalteu method was used. This involves reacting the sample with a reagent that forms a blue-coloured compound in the presence of phenolic compounds. The intensity of the blue colour is proportional to the amount of phenolic compounds present in the sample. To standardise the results, the TPC is expressed as the amount of gallic acid present in milligrams per milligram of extract (mg GAE/mg extract). This allows the results to be easily compared between samples, as the TPC is normalized to the amount of extract used. To determine the total flavonoid content (TFC) of a sample, the aluminium chloride (AlCl_3_) colorimetric method was used. This involves reacting the sample with a solution of aluminium chloride, which forms a yellow-coloured compound in the presence of flavonoids. The intensity of the yellow colour is proportional to the amount of flavonoids present in the sample. To standardise the results, the TFC is expressed as the amount of quercetin present in milligrams per milligram of extract (mg QE/mg extract). This allows the results to be easily compared between samples, as the TFC is normalised to the amount of extract used.

### Statistical analysis

All data were reported as the means of experiments conducted in triplicate. In these investigations, the significance of differences between tested extract was evaluated using one-way analysis of variance (ANOVA), followed by the Tukey test for further multiple comparisons, where probability (P ≤ 0.05) was indicated significant.

## Results

### Antioxidant activities

This study investigates the IC_50_ values of CBL-P and individual plant extract (*A. galangal*, *C. aurantifolia*, *T. triandra*, *C. sativa*, and *P. nigrum*) through the use of the DPPH and ABTS assays. Our results showed that CBL-P possessed antioxidant capacity with the IC_50_ of 8.549 ± 0.241 mg/mL and 2.673 ± 0.437 mg/mL for DPPH and ABTS assays, respectively (Table 1). However, the IC_50_ values of CBL-P was not as potent as ascorbic acid and Trolox which were used as a positive control.

**Table 1:** The IC_50_ values of CBL-P and each plant extracts using DPPH and ABTS assays

Samples	DPPH assay IC_50_ (mg/mL)	ABTS assay IC_50_ (mg/mL)
CBL-P	8.549 ± 0.241	2.673 ± 0.437
*A. galangal*	9.871 ± 0.145	6.384 ± 0.242
*C. aurantifolia*	12.323 ± 0.321	9.912 ± 0.276
*T. triandra*	0.096 ± 0.112	0.058 ± 0.107
*C. sativa*	0.392 ± 0.287	0.132 ± 0.206
*P. nigrum*	0.214 ± 0.121	0.125 ± 0.115
Ascorbic acid	0.040 ± 0.216	-
Trolox	-	0.069 ± 0.328

The values are represented as average mean ± SEM

### Inhibition of nitric oxide (NO) production

**Fig. 1:**
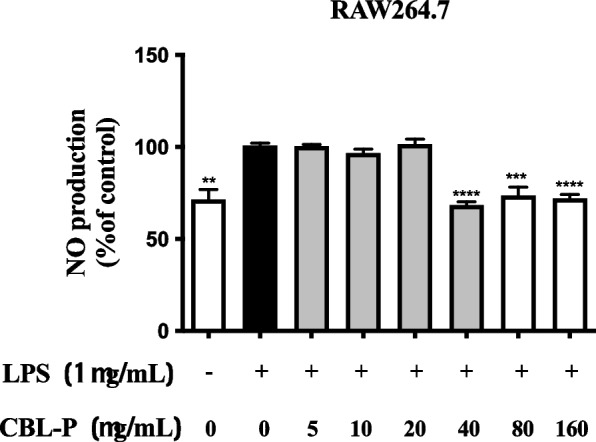
The effect of CBL-P on NO production. CBL-P the concentration of 40, 80, 160 μg/mL significantly inhibited NO production. Data were represented as mean ± SEM values of 3 independent tests (*n* = 3), with each experiment completed in triplicate. **P ≤ 0.01, ***P ≤ 0.001 and ****P ≤ 0.0001 compared to the control

NO is an inflammatory mediator generated by activated RAW264.7 cells which was induced by LPS. NO was measured using Griess reagent in nitrite ions (NO_2_^−^) form in cell culture medium. At 24 h, NO production was significantly decreased by CBL-P extract at 40, 80, 160 μg/mL. The inhibitory activity of CBL-P on NO production by induced RAW 264.7 macrophage cell lines is presented in Fig. 1. CBL-P extract had the best inhibitory activity on NO production (98.23% inhibition and 96.61% cell viability) at 40 μg/mL **(**Table 2). In addition, the potential to inhibit NO production of CBL-P from 40 to 160 μg/mL were significantly different compared to the control. Furthermore, CBL-P up to 40 μg/mL did not exhibit cytotoxicity on RAW264.7 cells and CBL-P from 80 to 160 μg/mL showed little cytotoxic activity on RAW264.7 cells.

**Table 2:** Inhibitory activity of CBL-P on the LPS-induced NO production in RAW 264.7 cells

**CBL-P (μg/mL)**	**% NO inhibition**	**% Cell viability**
**0**	0.45 ± 0.912	99.32 ± 0.562
**10**	15.68 ± 0.106	97.78 ± 0.362
**20**	30.39 ± 0.154	98.23 ± 0.429
**40**	98.24 ± 0.273^*^	96.61 ± 0.337
**80**	89.25 ± 0.851^*^	90.32 ± 0.462^*^
**160**	91.87 ± 0.347^*^	85.86 ± 0.571^*^

Data were expressed as mean ± SEM values of 3 independent experiments (*n* = 3), with each experiment completed in triplicate. *P ≤ 0.05 compared to the control

### Cytotoxic activity and antiproliferative activity

**Fig. 2:**
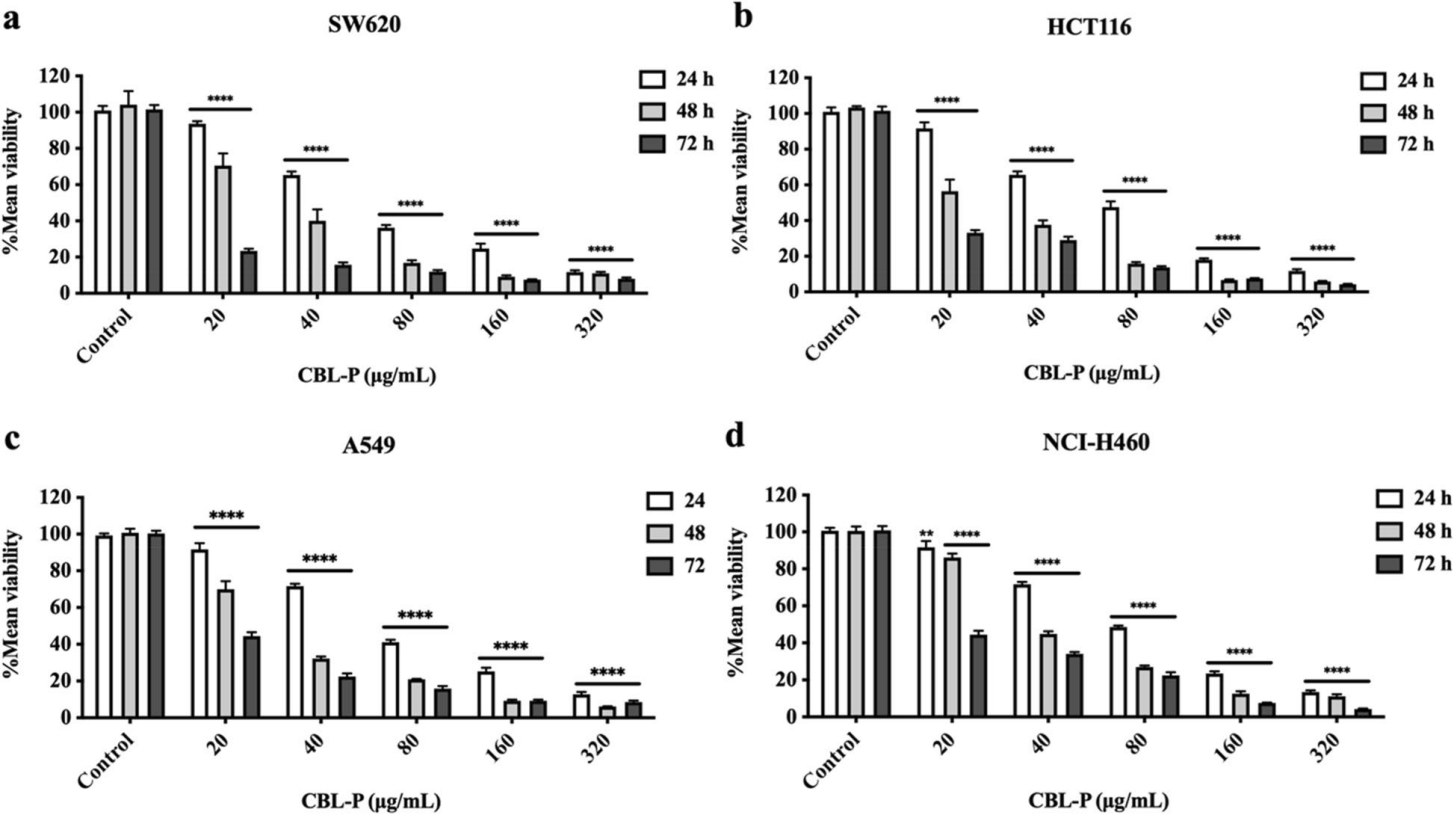
The cytotoxic activity of CBL-P on SW620, HCT116, A549, and H460 cell lines. The percentage viability of SW620 (**a**), HCT116 (b), A549 (**c**), and H460 (**d**) cell lines after 24, 48, and 72 h of CBL-P treatment. Data were expressed as mean ± SEM values of 3 independent tests (*n* = 3), with each experiment completed in triplicate. **P ≤ 0.01 and ****P ≤ 0.0001 compared to the control

This study assessed the efficacy of CBL-P in suppressing cell growth. Figure 2 showed the antiproliferative activity of CBL-P on four studied cell lines. Before conducting the cytotoxic activity of CBL-P on cancer cells, the effect of vehicle control (0.2%DMSO in completed medium) on cell viability of all studied cell lines were performed to ensure that the results of cytotoxicity of CBL-P were not interfered by the effect of vehicle control. The results illustrated that the vehicle control was not detrimental the growth of cancer cells and can be utilised safely for dissolving CBL-P.

CBL-P inhibited the growth of SW620, HCT116, A549, and NCI-H460 cell lines, as shown by the reduction of percentage of cell viability after the treatment of CBL-P for 24, 48, and 72 h. In this assay, our results showed that the percentage viability of SW620, HCT116, A549, and NCI-H460 significantly decreased after treated with CBL-P at increasing concentration (0–320 μg/mL) as a concentration-dependent manner (Fig. 2a - d). In addition, CBL-P also showed the antiproliferative activity as time- dependent manners with distinct IC_50_ values of four studied cell lines in each incubation time (24, 48, and 72 h) (Table 3). Interestingly, at the same incubation time the IC_50_ value of CBL-P was not significantly different between colorectal cancer (SW620 and HCT116) and non-small cell lung cancer cell (A549 and NCI-H460). This anticancer effect of CBL-P could be due to the molecular action of CBL-P could be similar effects on each cancer cell line. The cytotoxic activity of CBL-P, a mixture extract containing *A. galanga, P. nigrum, C. aurantifolia, T. triandra, and C. sativa*, was more potent compared to individual plant extracts (Table 4).

**Table 3:** The IC_50_ of CBL-P on SW620, HCT116, A549, and H460 cell lines at 24, 48, and 72 h

**Time (h)**	**CBL-P IC**_**50**_** (µg/mL) ± SEM**			
	**SW620**	**HCT116**	**A549**	**NCI-H460**
**24**	71.32 ± 0.782	62.48 ± 0.891	69.92 ± 1.021	75.26 ± 1.352
**48**	32.92 ± 1.394^*^	25.12 ± 1.065^*^	30.05 ± 1.284^*^	35.98 ± 2.113^*^
**72**	12.15 ± 0.654^*#^	10.57 ± 1.314^*#^	16.82 ± 0.725^*#^	19.72 ± 0.871^*#^

The values represented as mean IC_50_ ± SEM, *n* = 3; ^*^P ≤ 0.05 and ^#^*P* ≤ 0.05 compared to each particular cell at 24 and 48 h incubation time, respectively

**Table 4:** The comparison of IC_50_ of CBL-P and individual plant extract (*A. galangal*, *C. aurantifolia*, *T. triandra*, *C. sativa*, and *P. nigrum*) on SW620, HCT116, A549, and H460 cell lines at 48 h

Extracts	IC_50_ (µg/mL) ± SEM at 48 h			
	**SW620**	**HCT116**	**A549**	**NCI-H460**
**CBL-P**	32.92 ± 1.394^a,b,c,d,e^	25.12 ± 1.065 ^a,b,d,e^	30.05 ± 1.284 ^b,c,d,e^	35.98 ± 2.113 ^b,c,d,e^
***A. galangal***	38.26 ± 1.015	41.98 ± 0.822	32.32 ± 2.241	36.84 ± 0.924
***C. aurantifolia***	124.73 ± 0.942	95.91 ± 0.72	187.26 ± 1.845	198.22 ± 2.315
***T. triandra***	15.12 ± 1.224	26.56 ± 1.034	35.12 ± 2.034	39.56 ± 1.151
***C. sativa***	57.83 ± 1.715	33.89 ± 1.167	37.14 ± 2.101	29.95 ± 1.970
***P. nigrum***	62.78 ± 2.036	68.22 ± 2.124	58.32 ± 1.902	64.78 ± 1.116

The values represented as mean IC_50_ ± SEM, *n* = 3; ^a^*P* ≤ 0.05, ^b^*P* ≤ 0.05, ^c^*P* ≤ 0.05, ^d^*P* ≤ 0.05, and ^e^*P* ≤ 0.05 compared to the extracts of *A. galangal, C. aurantifolia, T. triandra, C. sativa, and P. nigrum* at 48 h, respectively, for each particular cell line

### Antimigration on cancer cell lines

**Fig. 3:**
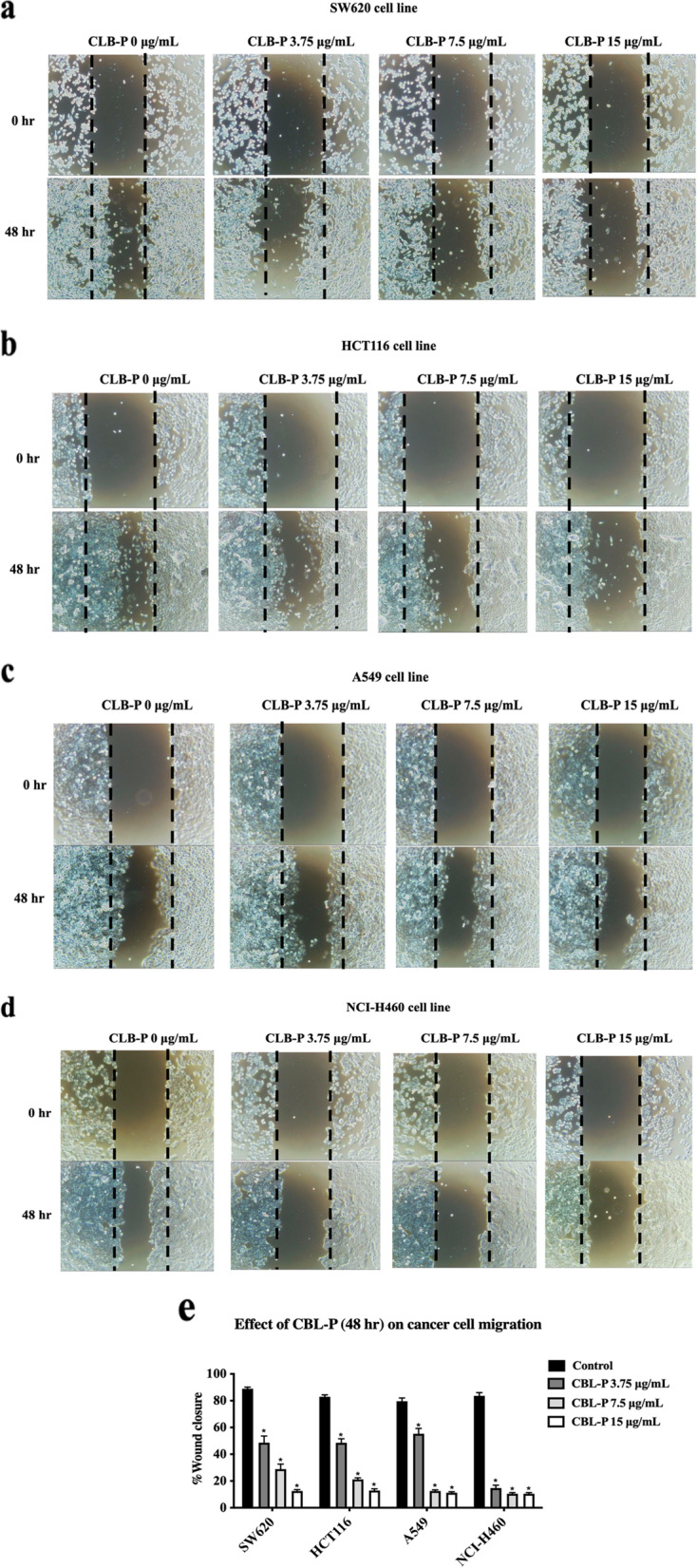
The antimigration effect of CBL-P on SW620, HCT116, A549, and NCI-H460 cell lines. The wound closure area of colorectal cancer SW620 (**a**), colorectal cancer HCT116 (**b**), non-small cell lung cancer A549 (**c**), and non-small cell lung cancer NCI-H460 (**d**) after treatment with CBL-P at 3.75, 7.5, and 15 μg/mL for 48 h. The percentage wound closure of the studied cell lines (**e**). Data were represented as mean ± SEM values of 3 independent tests (*n* = 3), with each experiment completed in triplicate. *P ≤ 0.05 compared to the control

The wound healing experiment was done to assess the influence of CBL-P on the migration of cancer cells. The concentration used in this experiment was based on the concentration acquired from the viability assay. The non-toxic concentrations were selected based the IC_50_ value of CBL-P at 48 h treatment. To ensure the safety of the experiment, the concentration of CBL-P used was selected to be less than the IC_50_ value of approximately 30 µg/mL at 48 h, as determined by cytotoxicity testing on four cell lines. The chosen concentration was achieved through a two-fold dilution process, resulting in a cell viability of greater than 70% for all cell lines [38]. This is in line with the INTERNATIONAL ISO STANDARD 10,993–5, which specifies that if the reduction in viability of the cell culture with the highest concentration of a sample extract is 30% or less, the material can be considered non-cytotoxic [39]. In other words, the ISO standard suggests that a chemical or substance can be considered non-toxic if it does not cause more than a 30% reduction in the viability of cells in culture when exposed to the highest concentration of the sample. By selecting non-toxic concentrations of CBL-P that resulted in a viability of all cell lines higher than 70%, this study aimed to ensure that the extract did not cause significant harm to the cells and could be studied for its effects on cancer cell migration. Three non-toxic concentration (3.75, 7.5, and 15 μg/mL) were used in this experiment since the concentrations did not cause major cell death which could interfere the interpretation of the results. Figure 3a - d depicted the movement of SW620, HCT116, A549, and NCI-H460 cell lines with the exposure of CBL-P during wound healing process. After 48 h of CBL-P incubation, wells containing CBL-P at 3.75, 7.5, and 15 μg/mL exhibited antimigration activity on A549 cell line with a per cent closure area of 53.12, 41.25, and 10.23%, respectively. While the per cent would closure of CBL-P on NCI-H460 at 3.75, 7.5, and 15 μg/mL were 15.21. 11.94, and 9.16%, respectively. In addition, CBL-P (at 3.75, 7.5, and 15 μg/mL) showed similar potential on antimigration activity in both HCT116 and SW620 colorectal cancer cells (range of per cent closure area varies from 10 – 50%). Interestingly, CBL-P showed better antimigration efficacy on an aggressive NCI-H460 than A549 (Fig. 3e).

### Total phenolic and total flavonoid contents

Total phenolic content (TPC) and total flavonoid content (TFC) are two commonly used measures of the antioxidant activity of plant-based compounds. TPC and TFC are measures of the concentration of phenolic compounds and flavonoids present in a sample, respectively. These compounds are known to have a number of biological activities, including antioxidant, anti-inflammatory, and anti-carcinogenic effects. The TPC of CBL-P was 648.19 mg GAE/mg extract. The TFC of CBL-P was 265.82 mg QE/mg extract (Table 5).

**Table 5:** Total phenolic content (TPC) and total flavonoid content (TFC) of CBL-P extract

Extract	TPC (mg GAE/mg extract)	TFC (mg QE/mg extract)
CBL-P	648.19 ± 0.365	265.82 ± 0.729

## Discussion

Herbal remedies have been used to maintain health and treat diseases in many places worldwide for a long time [16]. However, they are not officially recognised internationally because qualitative and quantitative evidence on their safety and effectiveness is insufficient [15, 16]. Herbal medications used in traditional folk medicine have long played a significant role in rural and urban health care in various nations, including Thailand [16, 40]. CBL-P is a mixture extract that consists of *A. galanga, P. nigrum, C. aurantifolia, T. triandra, and C. sativa.* These plants contain a variety of phytochemicals with potential health benefits. *A. galanga* is known for its antioxidant, anti-inflammatory, and anticancer properties and has been used traditionally to treat a variety of ailments. *P. nigrum* has antioxidant, anti-inflammatory, and analgesic properties and has been traditionally used to treat a variety of conditions. *C. aurantifolia* has antioxidant, anti-inflammatory, and antibacterial properties and has been traditionally used to treat a variety of conditions. *T. triandra* has antioxidant, anti-inflammatory, and hepatoprotective properties and has been traditionally used to treat a variety of ailments. *C. sativa* has a variety of potential health benefits including pain relief, anxiety reduction, and anti-inflammatory effects and has been used for medicinal and recreational purposes. The combination of these plants in CBL-P may have synergistic effects on various biological processes. Antioxidants neutralise reactive oxygen species, hydroxyl radicals, and nitric oxide, whereas anti-inflammatory mediators modulate the activities of pro-inflammatory enzymes and cytokines [41, 42]. An accumulation of free radicals causes damage to cells and is suspected to be the underlying cause of a wide variety of disorders. In the present study, we explored multiple biological activities of mixture extract of six Thai traditional medicine Clears Belong Plus (CBL-P) which consisted of *A. galanga*, *P. nigrum*, *C. aurantifolia*, *T. triandra*, and *C. sativa*. The antioxidative analysis methods were carried out and the results showed that CBL-P possessed substantial antioxidant potential. It is speculated that the hydrogen-donating capability of the test extracts is responsible for the effect those extracts had on DPPH and ABTS radicals scavenging. Also, the anti-inflammatory activity was conducted in RAW264.7 cells after the safety testing of CBL-P on RAW264.7 cells via cytotoxicity assay. CBL-P did not exhibit any cytotoxic effects at concentrations of up to 320 μg/mL. Thus, the anti-inflammatory effect of a non-toxic concentration (0–160 μg/mL) was subsequently determined for anti-inflammatory activity. CBL-P showed promised activity on anti-inflammatory activity by inhibiting NO production with no significant toxicity to the cells **(**Table 2**)**. These results suggested that the bioactive constituents of CBL-P had both antioxidant and anti-inflammatory activities. CBL-P with effective inhibition of NO generation and minimal cytotoxicity is more beneficial. To lessen the inflammation caused by nitric oxide (NO) release, extracts that have the potential to act as NO scavengers or production inhibitors, especially those with low cytotoxicity, could be used. The reduction of NO generation by extracts from medicinal plants may be related to the inhibition of the activity or overexpression of inducible nitric oxide synthase [4]. The suppression of NO generation by medicinal plant extracts may be related to the suppression of inducible nitric oxide synthase activity or its expression.

**Fig. 4:**
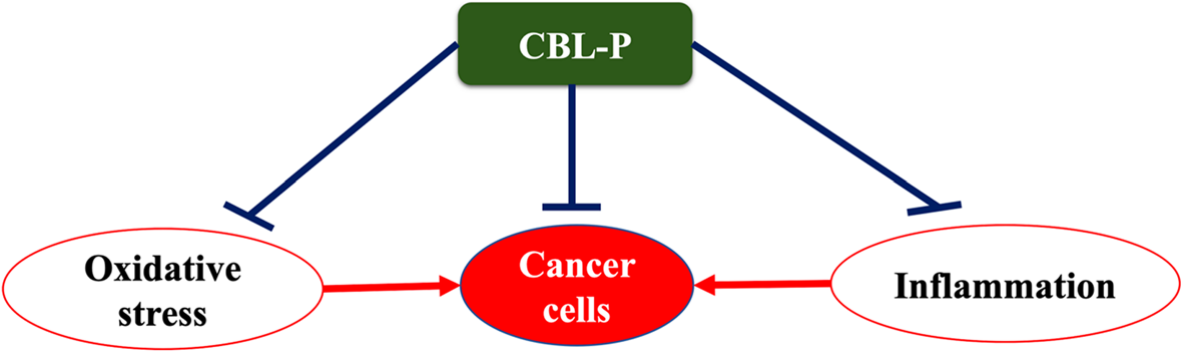
Proposed anticancer mechanism of CBL-P

Furthermore, our study indicated that CBL-P exhibited strong cytotoxic and antiproliferative effects against colorectal cancer HCT116 and SW620 as well as non-small cell lung cancer A549 and NCI-H460 cells as a concentration- and time-dependent manner. The observation that CBL-P has more potent cytotoxic activity than individual plant extracts suggests that the combination of these plants may have a synergistic effect on cell growth and survival. This means that the combination of these plants may be more effective at inhibiting the growth of cancer cells than any of the individual plants alone. Interestingly, this CBL-P was a mixture extract of plants which exert significant antimigration activity at very low concentration. Several researches have been conducted on a wide variety of plants to determine the levels of phenolic and flavonoid chemicals they contain and the effects these compounds have on cancer cells. The cytotoxicity of polyphenols on various cancer cell lines has been proven, and the antioxidant capabilities of polyphenols have been explored [43]. It takes longer to produce significant quantities of plant secondary metabolites because of their poor solubility or poor bioavailability, both limiting traits connected to many plant secondary metabolites. These metabolites are needed to function as therapeutic medications. By using synergistic effect of medicinal plants would be one of the solutions to solubility issue. Our results indicated that CBL-P as a promising anticancer compound that can inhibit proliferation antimigration of multiple cancer cells could be due to their synergistic effects of phytochemical constituents. Furthermore, CBL-P, with the combination of Thai medicinal plants, enables the development of a wide range of cancer treatments and possibly other pathological conditions and personalised treatments. Therefore, our finding suggested that anticancer activity of the CBL-P could strongly attributed to their promised antioxidant and anti-inflammatory activities (Fig. 4**)**. However, further investigation on molecular level of CBL-P on anticancer mechanism related inflammatory cytokines and signalling are required.

CBL-P is a mixture extract that contains high amount of phenolics and flavonoids. These types of phytochemicals are known to have multiple biological activities. The presence of high levels of phenolic and flavonoid compounds in CBL-P suggests that it may have antioxidative, anti-inflammatory, and anticancer activities. The inclusion of these compounds in CBL-P may contribute to its overall therapeutic potential, making it a promising adjuvant compound for cancer treatment. However, more research is needed to fully understand the mechanisms of action and potential therapeutic applications of CBL-P.

## Conclusion

This study demonstrated that CBL-P has significant anticancer potential. In addition, the data given in this study reveals the antioxidant and anti-inflammatory actions of CBL-P, which correspond to the ethnomedical claims made for each plant species in a mixed extract. The mechanisms responsible for the anti-inflammatory properties of the CBL-P ethanolic extract appear to be associated with its ability to inhibit NO generation and serve as a free radical scavenger. In addition, we demonstrate for the first time that CBL-P antioxidant effectiveness and anti-inflammatory activity may be correlated to its anticancer effects. The overall biological properties of CBL-P, a mixture extract containing five medicinal plants, were demonstrated to have anticancer potential in this study. In addition, CBL-P was found to possess antioxidant and anti-inflammatory activity, as demonstrated by its ability to inhibit the production of NO and serve as a free radical scavenger. These findings suggest that the phenolics and flavonoids present in CBL-P, may provide therapeutic and health-promoting benefits. The precise mechanisms by which these compounds contribute to the therapeutic and health-promoting effects of CBL-P are not yet fully understood and require further investigation. However, these findings suggest that CBL-P may be a promising adjuvant compound for cancer therapeutic purposes. Regarding the overall biological properties of CBL-P, it could be regarded as the primary bioactive compound responsible for synergistic medicinal plants' therapeutic and health-promoting potential. It could be used as a promising adjuvant compound for cancer therapeutic purposes.

## Data Availability

The datasets used and/or analysed during the current study available from the corresponding author on reasonable request.
